# Synergistic Effects of Kaolin and Silicon Nanoparticles for Ameliorating Deficit Irrigation Stress in Maize Plants by Upregulating Antioxidant Defense Systems

**DOI:** 10.3390/plants12112221

**Published:** 2023-06-05

**Authors:** Alshymaa Z. Al-Mokadem, Mohamed H. Sheta, Ahmed G. Mancy, Hebat-Allah A. Hussein, Sahar K. M. Kenawy, Ahmed R. Sofy, Mahmoud S. Abu-Shahba, Hesham M. Mahdy, Mahmoud R. Sofy, Alaa Fathy Al Bakry, Mona S. Agha

**Affiliations:** 1 Botany Department, Women’s College, Ain Shams University, Cairo 11566, Egypt; 2 Chemistry Department, College of Science and Arts, Jouf University, Al-Gurayyat 77447, Saudi Arabia; 3 Soils and Water Department, Faculty of Agriculture, Al-Azhar University, Cairo 11884, Egypt; 4 Botany and Microbiology Department, Faculty of Science (Girls Branch), Al-Azhar University, Cairo 11754, Egypt; 5 Biology Department, University College of Nairiyah, University of Hafr Al-Batin, Nairiyah 31991, Saudi Arabia; 6 Botany and Microbiology Department, Faculty of Science, Al-Azhar University, Cairo 11884, Egypt; 7 Al-Azhar Center for Fermentation Biotechnology and Applied Microbiology, Al-Azhar University, Cairo 11884, Egypt; 8 Soil Fertility and Plant Nutrition Department, Soil Water and Environment Research Institute, Agriculture Research Center, Giza 12619, Egypt; 9 Botany Department, Faculty of Science, Mansoura University, Mansoura 35516, Egypt

**Keywords:** drought, enzymatic and non-enzymatic antioxidants, yield, ROS indicator and mineral content

## Abstract

Water deficit is a significant environmental stress that has a negative impact on plant growth and yield. In this research, the positive significance of kaolin and SiO_2_ nanoparticles in moderating the detrimental effects of water deficit on maize plant growth and yield is investigated. The foliar application of kaolin (3 and 6%) and SiO_2_ NPs (1.5 and 3 mM) solutions increased the growth and yield variables of maize plants grown under normal conditions (100% available water) and drought stress conditions (80 and 60% available water (AW)). In addition, plants treated with SiO_2_ NPs (3 mM) demonstrated increased levels of important osmolytes, such as proline and phenol, and maintained more of their photosynthetic pigments (net photosynthetic rate (PN), stomatal conductance (gs), intercellular CO_2_ concentration (Ci), and transpiration rate (E)) than with other applied treatments under either stress or non-stress conditions. Furthermore, the exogenous foliar application of kaolin and SiO_2_ NPs also reduced the amounts of hydroxyl radicals (OH), superoxide anions (O_2_), hydrogen peroxide (H_2_O_2_), and lipid peroxidation in maize plants experiencing a water deficit. In contrast, the treatments led to an increase in the activity of antioxidant enzymes such as peroxidase (POX), ascorbate peroxidase (APX), glutathione peroxidase (GR), catalase (CAT), and superoxide dismutase (SOD). Overall, our findings indicate the beneficial impact of the application of kaolin and silicon NPs, particularly the impact of SiO_2_ NPs (3 mM) on managing the negative, harmful impacts of soil water deficit stress in maize plants.

## 1. Introduction

Water scarcity is a key growth-limiting factor for agricultural production which may provide a significant barrier to fulfilling global food security and environmental sustainability goals [1]. Nowadays, water shortages affect 40–60% of the world’s agricultural area [2]. According to Wudil et al. [3], the primary contributors to water scarcity include the unequal distribution of water resources, a fast-expanding population and urbanization, and industrialization. In addition, water shortages may change the biochemical and physiological processes inside plants, significantly decreasing plant growth and production [4]. The main mechanisms responsible for decreased plant productivity under water stress may include reduced seed germination, decreased leaf growth [5], the inhibition of photosynthesis, reduced water and ion uptake, the inactivation of enzymes [6], membrane destabilization [7], an increase in the production of reactive oxygen species [8], flower shedding, and yield loss [9]. Furthermore, oxidative stress is one of the main processes that cause a reduction in the development and production of many crop species in stressful environments [10]. Hanif et al. [11] also showed that the key indicators for determining a plant’s tolerance to numerous abiotic and biotic stresses such as water shortage are osmotic stress, oxidative damage, lipid peroxidation, which is measured in terms of malondialdehyde (MDA), the disruption of photosynthetic pigments, and photosynthesis.

In order to utilize water more efficiently, effective advances in irrigation and management approaches are required [12]. In this regard, deficit irrigation (DI), which involves providing less water than is necessary for irrigation, is utilized [13].

A protective particle coating may be created by spraying kaolin mineral particles, which are hydrated aluminum silicates (AL_2_Si_2_O_5_ (OH)_4_), over the surface of leaves. This induces the reflection of light on the leaf’s surface, which lowers the leaf temperature and prolongs stomatal opening when the deficit in air vapor pressure is significant [14]. Recently, agricultural crops have used particle film technology with inert reflecting materials such as kaolin to minimize heat and water stress. It has also been utilized for insect management and disease incidence reduction.

Recently, interest has grown in using nanomaterials in various industries, including nano fertilizers. A nanoparticle’s small size (<100 nm), which results in a large surface charge and area, creates new properties and makes it more reactive than a bulk-scale particle [15]. These nanoscale fertilizers are a technology that increases the efficiency of plant nutrient absorption by making the nutrients accessible to plant leaves [16]. According to reports, SiO_2_ NPs may reduce oxidative damage in a variety of crops [17]. For example, SiO_2_ NPs decreased malondialdehyde (MDA) levels in stressed plants and increased the levels of CAT and SOD activity [18]. The advantage of nanoscale fertilizers is that they can reduce the rate of nutrient addition, save input costs, and reduce the environmental impact of chemical fertilizers [19]. In addition, nano fertilizers demonstrate greater and quicker translocation between plant parts due to their tiny size, which boosts the utilization of nutrients [20]. Traditional Si fertilizer in the form of Na_2_SiO_3_ has a very limited Si usage efficiency (1–5%). In contrast, silicon oxide nanoparticles (SiO_2_ NPs) are being carefully studied in plants to improve their capacity to control crop yield and nutrient usage efficiency [21]. The use of nano fertilizers has been demonstrated to improve the drought tolerance of a number of crops, including strawberries [22] and wheat [23]. For example, SiO_2_ NPs were sprayed on wheat plants to promote growth, antioxidant activity, and chlorophyll content according to Akhtar et al. [21]. SiO_2_ NPs increased the total N and K absorption and green production in drought-stressed pea plants by up to 183% [24]. 

Maize (*Zea mays* L.) is one of the most significant cereal grains in the world because of its enhanced adaptability to various environments [25]. This raw material is primarily used as a food source and has evolved into one of the most efficient raw materials for food and feed [26]. It may also be utilized to generate bioenergy [27]. In addition, maize grains have significant nutritional value, and their oil is used in cooking. In 2018, the worldwide planted area of *Zea mays* was 193.7 Mha, with a total grain production of 1147.6 Mt. Egypt’s farmed maize acreage was roughly 0.94 Mha, with a total yield of 7.30 Mt 35. However, *Zea mays* is a C4 plant that is rated moderately sensitive to drought stress. Moreover, when grown under drought stress, its growth and yield can suffer significantly [28].

Little information is available on the applications of kaolin (commercial kaolin clay) and nano Si in alleviating drought-induced injuries in maize plants. This research was carried out in light of the protective abilities offered by Si in mitigating the unfavorable consequences of drought stress. Therefore, the main goal of this study was to gain fundamental knowledge about the effects of kaolin (a traditional salt of Si) and SiO_2_ NPs in modifying some morphological, physiological, and molecular attributes of maize plants exposed to a water deficit. This research was carried out in light of the protective abilities provided by Si in mitigating the unfavorable consequences of drought stress. 

Thus, foliar applications of kaolin and SiO_2_ NPs will provide insights into the mechanisms of plant tolerance under the conditions of a water deficit. Furthermore, the findings of this study will generate new research prospects for water deficit stresses in climate change scenarios.

## 2. Results

### 2.1. Growth and Yield Traits

**Figure 1:**
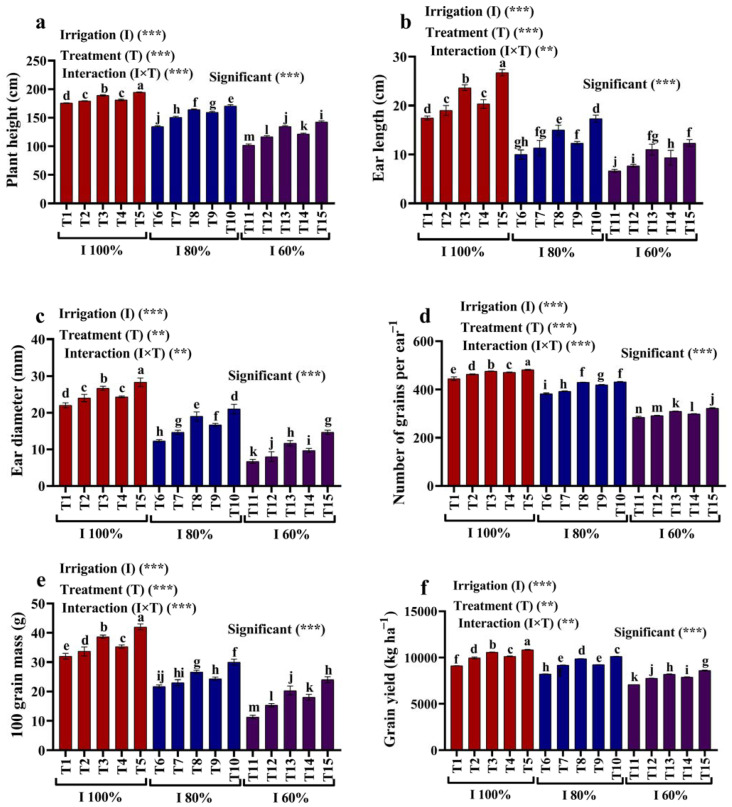
Effects of foliar applications of kaolin and SiO_2_ NPs on (**a**) plant height, (**b**) ear length, (**c**) ear diameter, (**d**) number of grains per ear, (**e**) 100-grain weight, and (**f**) grain yield of maize grown with available water (100%, 80%, and 60% AW). Fisher’s multiple range test indicates significant differences between means in each bar (*p* < 0.05). ** and *** indicate differences at *p* < 0.05 and *p* < 0.01 probability levels, respectively.

Figure 1a–f demonstrate the growth characteristics and yield of the maize plants, such as plant height, ear length, number of grains per ear, ear diameter, grain yield, and the 100-grain weight when irrigation is applied (60, 80, and 100% AW) and when kaolin (3 and 6%) and SiO_2_ NPs (1.5 and 3.0 mM) are applied. In this respect, 80% and 60% of AW reduced plant height by 23.34% and 41.94%, respectively; ear length by 42.78%, and 61.85%, respectively; ear diameter by 43.94% and 69.70%, respectively; the number of grains per ear by 13.92% and 36.08%, respectively; the 100-grain weight by 32.29% and 64.58%, respectively; and the grain yield 9.79% and 22.35%, respectively, compared with 100% of AW. These growth traits were significantly improved by through treatment with kaolin (3% and 6%) and SiO_2_ NPs (1.5 and 3 mM), and these improvements were more pronounced when SiO_2_ NPs (3 mM) were used compared to the non-stressed plants. The exogenous application of kaolin and SiO_2_ NPs alleviated the adverse impacts of water deficit stress on maize growth in the sense that applying kaolin (3% and 6%), and SiO_2_ NPs (1.5 and 3 mM) to maize plants grown under stress due to a water deficit were significantly improved when compared to plants grown with 80% and 60% AW.

### 2.2. Photosynthetic Characteristics

**Figure 2:**
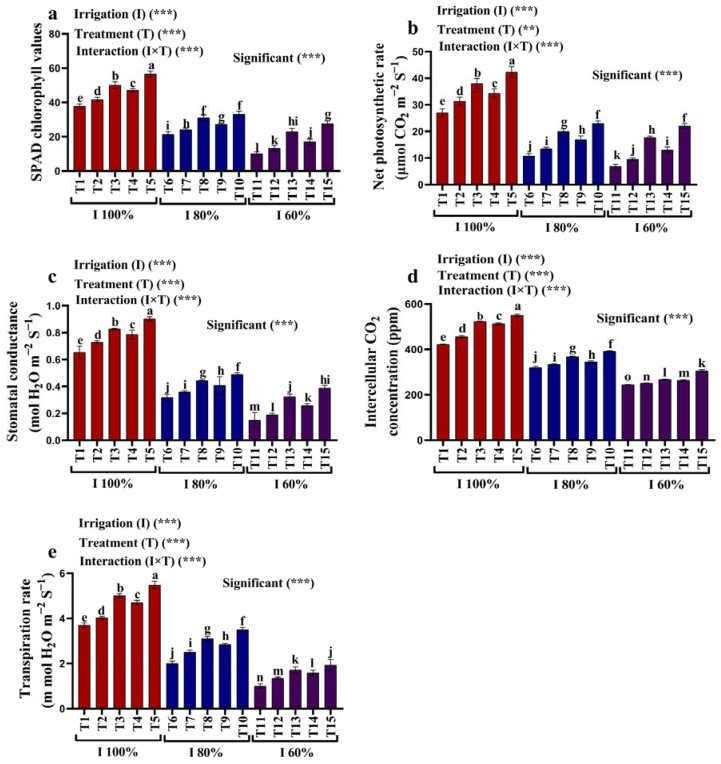
(**a**–**e**) Effects of foliar application of kaolin and SiO_2_ NPs on (**a**) SPAD chlorophyll values, (**b**) net photosynthetic rate, (**c**) stomatal conductance, (**d**) intercellular CO_2_ concentration, and (**e**) transpiration rate of maize grown under available water (100%, 80%, and 60% AW). Fisher’s multiple range test indicates significant differences between means in each bar (*p* < 0.05). ** and *** indicate differences at *p* < 0.05 and *p* < 0.01 probability levels, respectively.

Figure 2a–e show the effect of kaolin (3% and 6%) and SiO_2_ NPs (1.5 and 3 mM) on SPAD chlorophyll values and the photosynthetic characteristics (net photosynthetic rate (PN), intercellular CO_2_ concentration (Ci), stomatal conductance (gs), and transpiration rate (E)) in the leaves of maize plants experiencing water deficit stress. The SPAD chlorophyll (43.36%; 73.45%), PN (59.88%; 74.57%), gs (51.53%; 77.04%), Ci (24.11%; 42.13%), and E (45.95%; 72.97%) values were significantly decreased in the maize grown with irrigation with 80% and 60% of the AW when compared to the plants grown with full irrigation with 100% of the AW. In contrast, the photosynthetic properties and the contents of SPAD chlorophyll were increased in the maize plants treated with kaolin and SiO_2_ NPs compared to the plants grown with full irrigation with 100% of AW since the kaolin and the SiO_2_ NPs mitigated the adverse effects of the water deficit stress. The most effective concentration of 3 mM SiO_2_ NPs caused a significant increase in the SPAD chlorophyll (54.69%; 176.67%), PN (112.31%; 220.39%), gs (54.74%; 157.78%), Ci (22.60%; 25.00%), and E (75.00%; 92.67%) values when compared to irrigation with 80% and 60% of the AW, respectively (Figure 2a–e).

### 2.3. Lipid Peroxidation Content and ROS Production

**Figure 3:**
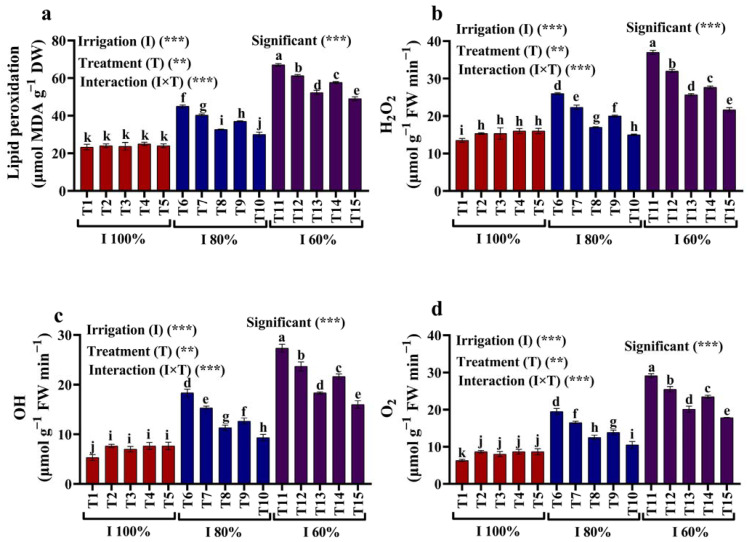
(**a**–**d**) Effects of foliar application of kaolin and SiO_2_ NPs on (**a**) lipid peroxidation (MDA), (**b**) hydrogen peroxide (H_2_O_2_), (**c**) hydroxyl radicals (OH), and (**d**) superoxide anion (O_2_) of maize grown with different amounts of available water (100%, 80%, and 60% AW). Fisher’s multiple range test indicates significant differences between means in each bar (*p* < 0.05). ** and *** indicate differences at *p* < 0.05 and *p* < 0.01 probability levels, respectively.

Regarding the outcomes of this experiment, some critical observations may be made for the development of MDA as an indicator of lipid peroxidation and ROS production (H_2_O_2_, OH, and O_2_) in maize leaves treated with kaolin (3 and 6%) and SiO_2_ NPs (1.5 and 3 mM) irrigated with 100%, 80%, and 60% of the AW (Figure 3a–d). Our findings demonstrate that allowing maize plants to grow under the stressful condition of a water deficit significantly increased their MDA, H_2_O_2_, OH, and O_2_ contents. Applying different concentrations of kaolin (3 and 6%) and SiO_2_ NPs (1.5 and 3 mM) proved their significant ability to alleviate water deficit stress by reducing the MDA, H_2_O_2_, OH, and O_2_ contents. The highly significant decrease in MDA, H_2_O_2_, OH, and O_2_ contents in all stressed plants were observed when using SiO_2_ NPs (3 mM).

### 2.4. Proline, Phenol, and Non-Enzymatic Antioxidants

**Figure 4:**
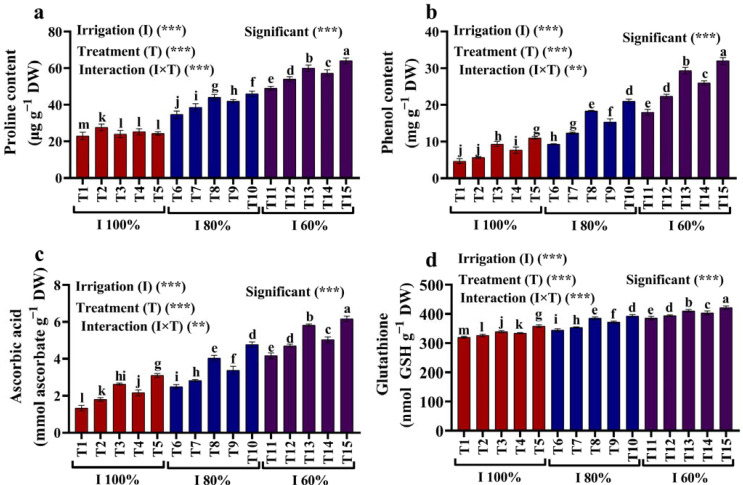
(**a**–**d**) Effects of foliar application of kaolin and SiO_2_ NPs on (**a**) proline, (**b**) phenol, (**c**) ascorbic acid, and (**d**) glutathione of maize grown with different amounts of available water (100%, 80%, and 60% AW). Fisher’s multiple range test indicates significant differences between means in each bar (*p* < 0.05). ** and *** indicate differences at *p* < 0.05 and *p* < 0.01 probability levels, respectively.

It is worth noting that the water deficit significantly increased the proline, phenol, and non-enzymatic antioxidant levels (Figure 4a–d). Nevertheless, kaolin and SiO_2_ NPs applied to water-deficit-stressed plants increased the proline, phenol, AsA, and GSH levels much more than in the stressed plants. In addition, when the maize plants were treated with 3.0 mM SiO_2_ NPs instead of 6% kaolin, there was a considerable increase in their proline, phenol, AsA, and GSH contents (Figure 4a–d).

### 2.5. Antioxidant Enzymes

**Figure 5:**
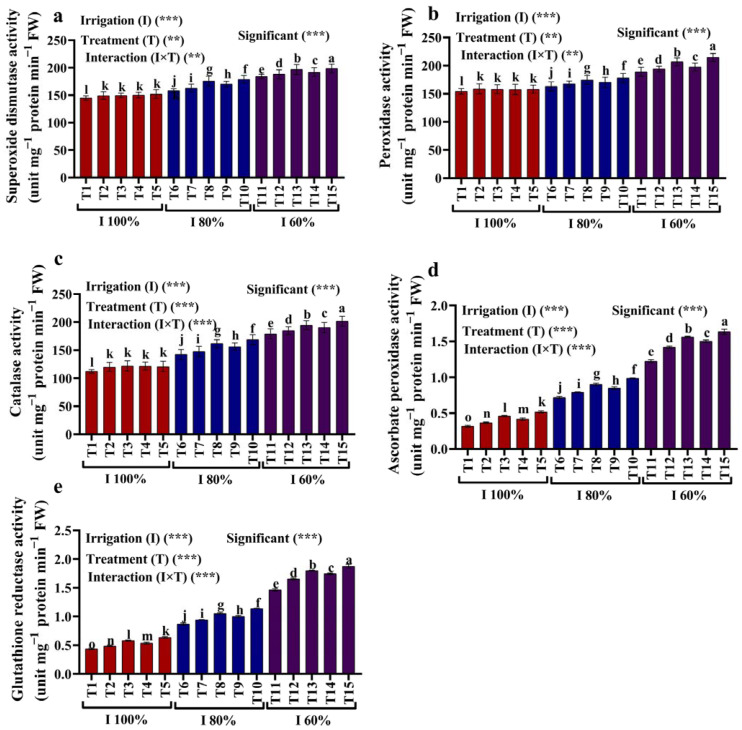
(**a**–**e**) Effects of foliar application of kaolin, SiO_2_ NPs on (**a**) superoxide dismutase activity (SOD), (**b**) peroxidase activity (POX), (**c**) catalase activity (CAT), (**d**) ascorbate peroxidase activity (APX), and (**e**) glutathione reductase activity (GR) of maize grown under different available water conditions (100%, 80%, and 60% of the AW). Fisher’s multiple range test indicates significant differences between means in each bar (*p* < 0.05). ** and *** indicate differences at *p* < 0.05 and *p* < 0.01 probability levels, respectively.

Water deficit increased the SOD, POX, CAT, APX, and GR activities compared to full irrigation with 100% of the AW (Figure 5a–e). The exposure to irrigation with 80% and 60% of the AW significantly increased the antioxidant activities of SOD (8.97%; 27.13%), POX (5.62%; 22.46%), CAT (26.71%; 59.35%), APX (126.32%; 286.32%), and GR (98.47%; 235.11%) compared to the plants grown with full irrigation with 100% of the AW. In addition, kaolin and SiO_2_ NPs stimulated the activities of the antioxidant enzymes POX, SOD, CAT, APX, and GR. For example, maize plants raised with the foliar application of 3 mM SiO_2_ NPs exhibited the highest SOD (13.29%, 7.78%), POX (9.20%, 13.58%), CAT (18.50%, 12.66%), APX (37.67%, 33.51%), GR (31.15%, 28.02%) activities in comparison to plants grown with irrigation with 80% and 60% of the AW, respectively.

### 2.6. Mineral Content

**Figure 6:**
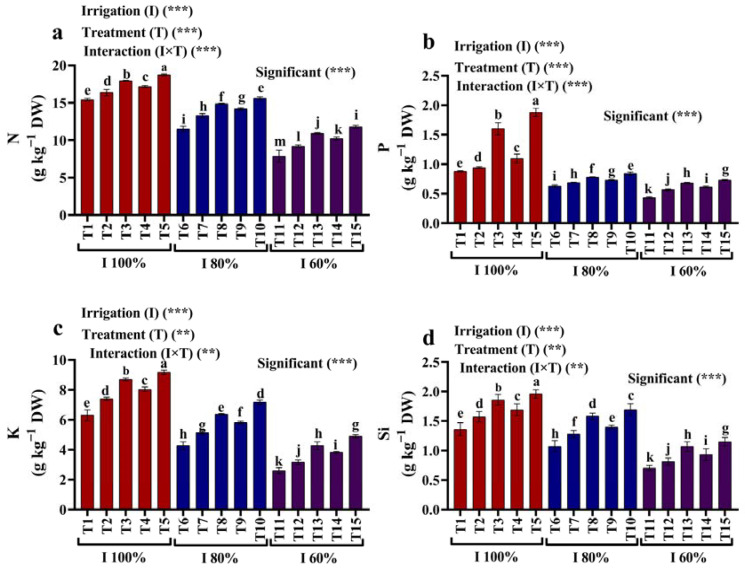
(**a–d**) Effects of foliar application of kaolin and SiO_2_ NPs on (**a**) nitrogen (N), (**b**) phosphorous (P), (**c**) potassium (K), and (**d**) silicon (Si) of maize grown under different available water conditions (100%, 80%, and 60% AW). Fisher’s multiple range test indicates significant differences between means in each bar (*p* < 0.05). ** and *** indicate differences at *p* < 0.05 and *p* < 0.01 probability levels, respectively.

Compared to the non-drought-stress plants, water deficit stress reduced the N, P, K, and Si contents of the maize grains (Figure 6a–d). Nevertheless, compared to plants cultivated with full irrigation with 100% of the AW, applying kaolin and SiO_2_ NPs considerably increased the maize grains’ N, P, K, and Si contents (Figure 6a–d). Compared to plants grown with irrigation with 80% and 60% of the AW, the exogenous treatment with 3 mM SiO_2_ NPs showed the most effective treatment effect.

### 2.7. A Principal Component Analysis and Heat Map PeA.R.S.on Correlation

**Figure 7:**
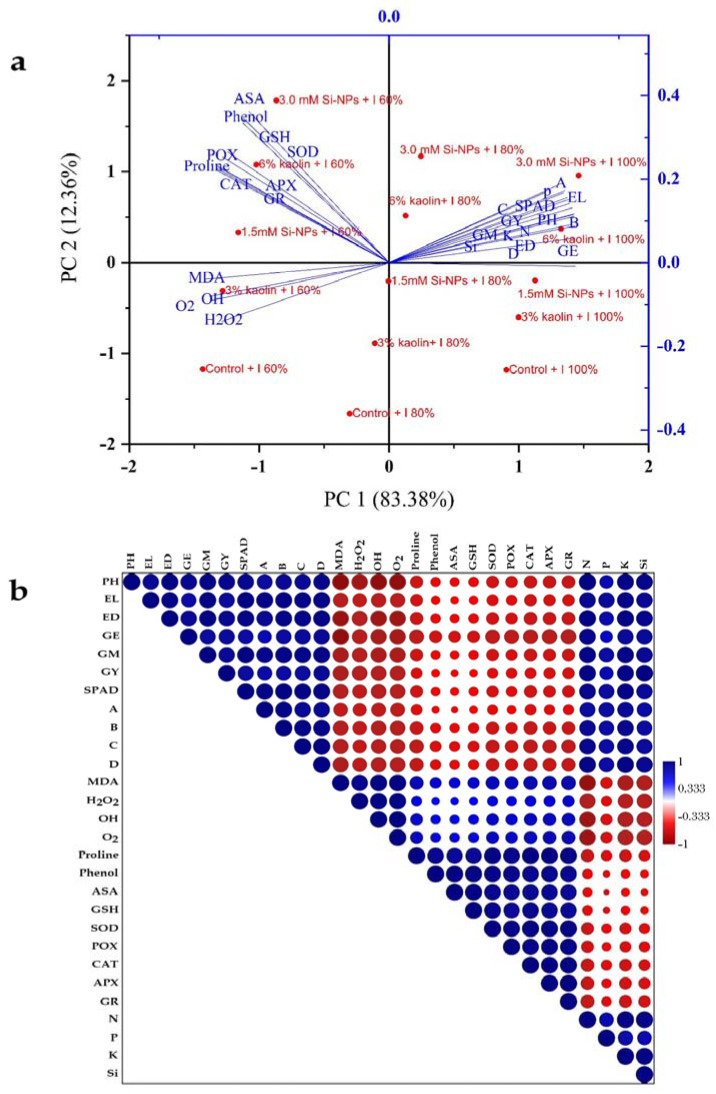
(**a**) An analysis of the correlations between treatment variables in maize plants using principal component analysis (PCA). (**b**) Based on the mean values of different parameters reported in this study, the heat map confirms the association between quantitative statistical parameters. PH; plant height, EL, ear length; ED, ear diameter; GE, number of grains per ear^−1^; GM, 100-grain mass; GY, grain yield; A, net photosynthetic rate; B, stomatal conductance; C, intercellular CO_2_ concentration; D, transpiration rate.

**Figure 8:**
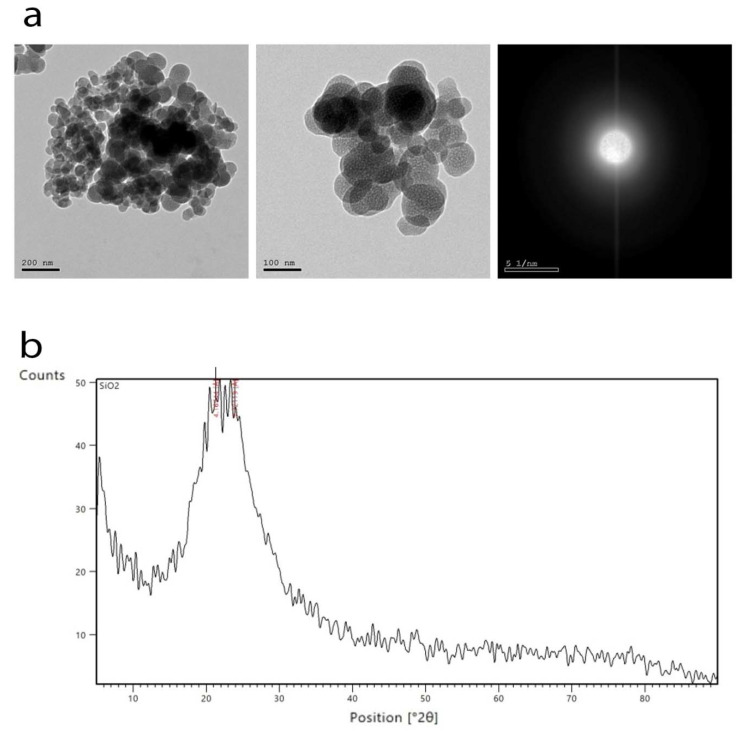
(**a**) TEM images of the prepared SiO_2_ NPs; (**b**) XRD pattern of the prepared SiO_2_ NPs.

A principal component analysis was performed on the different applications based on morpho-biochemical parameters and non-drought-stressed or drought-stressed conditions. The principal components explained 95.74% (83.34% and 12.4%) of the total variance (Figure 7a). The heat map Pearson correlation coefficients between the grain maize yield and related indicators, shown in in Figure 8b, were calculated to illuminate the effectual attributes of the interest relationship. Highly significant and positive correlation coefficients were obtained between the grain yield and growth parameters, photosynthetic characteristics, and mineral content. In contrast, negative and highly significant correlation coefficients were obtained between the grain yield and the production of ROS, lipid peroxidation content, proline, phenol, non-enzymatic antioxidants, and antioxidant enzymes (Figure 7b).

## 3. Materials and Methods

This study was conducted at the Faculty of Agriculture, Al-Azhar University, Cairo, Egypt, in the Experimental Farm of Soils and Water Department, using a drip irrigation system during the summer seasons of 2021 and 2022 (30°03′19.0″ N 31°19′10.0″ E). The 1st factor included irrigation levels at 100% (I 100%), 80% (I 80%), and 60% (I 60%) of the available water (AW); the 2nd factor included four concentrations of the foliar application of kaolin (AL_2_Si_2_O_5_ (OH)_4_, 3 and 6%) and SiO_2_ NPs (1.5 and 3.0 mM). An experiment was conducted with a complete randomized design (CRD). The TEM image and XRD of SiO_2_ NPs are shown in Figure 8a,b. 

Method of Preparing Silicon Nanoparticles (SiO_2_-NPs) and their Characterization:

- Chemicals:

Tetraethoxysilane (TEOS, 99%, Chem-Lab), absolute ethanol, and ammonia 25% (purchased from Meck, Germany) and distilled water were used in this work. The chemicals were employed without any further purification.

- Procedure:

Silica nanoparticles were synthesized via a modified sol–gel method [29].

- General Properties:

Appearance (color): white

Appearance (form): powder

Molecular weight: 60.08 g/mol

Phase: amorphous

- Characterization:

Size and shape: TEM was performed using a JEOL JEM-2100 high-resolution transmission electron microscope at an accelerating voltage of 200 kV. The TEM and XRD images of the SiO_2_-NPs are illustrated in Figure 8a,b.

Soil samples were collected from four soil layers (0–60 cm) for physical and chemical property determination using the standardized methods of Page et al. [30] and Klute and Dirksen [31] (Table 1). As shown in Table 2, the climatic information for the experimental location throughout the summer growing seasons was acquired from the Central Laboratory for Agricultural Climate, located at the Agricultural Research Center in Giza, Egypt.

### 3.1. Plant Materials and Agricultural Practices

A variety of single cross 10 (S.C.10) maize grains (*Zea mays* L.) were purchased from the Giza Agricultural Research Center in Egypt. The grains were carefully rinsed multiple times with distilled water after being sterilized for 2 min in a 1% sodium hypochlorite solution. They were then left outside for two hours before they were soaked in distilled water at room temperature for eight hours. During the 2021 and 2022 summer growing seasons, maize grains were sowed on 10th May and harvested on 23rd August. The experimental plot area (0.70 m row width × 15 m length) was irrigated via a drip irrigation system with a total area of 10.5 m^2^ per plot and 0.25 m between plants within rows. Two grains were sown per pit at a depth of 0.04 m and 0.05 m from the drip line. After the maize grains were sown, the experimental plots were drip-irrigated with one line and one dripper per pit, providing 4.00 L h^−1^. After 20 days of sowing, the maize plants were thinned to one plant per pit. The recommended agricultural practices for maize plants according to the Egyptian Ministry of Agriculture were followed during the growing season. Phosphorus (P), potassium (K), and nitrogen (N) fertilizers were applied to the maize as recommended (285 kg N ha^−1^: urea (46.5% N); 114 kg K ha^−1^: potassium sulfate (48% K_2_O); 357 kg ha^−1^ calcium superphosphate (15.5% P_2_O_5_). The different irrigation levels began 20 days from planting. Foliar kaolin and SiO_2_ NPs concentrations were applied 40 and 60 days after sowing (DAS)

### 3.2. Irrigation Water Applied (IWA)

The irrigation water applied (IWA) was computed based on the soil moisture content in the effective root zone for maize plants. As a result, the amount of irrigation water used for each treatment during irrigation was measured based on the value of the available water percentage in the effective root zone. The soil moisture content was determined by using a pressure plate apparatus at suction pressures of −0.33 bar (field capacity, $\theta_{\mathrm{FC}}$) and −15.00 bar (permanent wilting point, $\theta_{\mathrm{PWP}}$), as described by Klute and Dirksen [31], via soil samples collected by a soil auger from soil layers based on the effective root length for maize plants prior to each water addition. The available water was computed by subtracting the moisture content at the permanent wilting point from the moisture content at field capacity. The volume of water applied per irrigation level to each plot was computed by the equation:

(1) $$\mathrm{IWA}=\frac{{(\theta }_{\mathrm{FC}}{-\theta }_{\mathrm{PWP}})\times D\times A}{E_{a}}$$

where IWA is the irrigation water applied (m^3^), $\theta_{\mathrm{FC}}$ is the soil moisture content at field capacity (m^3^ m^−3^), $\theta_{\mathrm{PWP}}$ is the soil moisture content at the permanent wilting point (m^3^ m^−3^), D is the effective root zone depth (m), A is the surface area of each plot (m^2^), and $E_{a}$ is the irrigation efficiency, $E_{a}$ = 85%. 

The groups were divided into:
Group IT1Full irrigation with 100% of AW.T2Full irrigation with 100% of AW + foliar application of 3% kaolin.T3Full irrigation with 100% of AW + foliar application of 6% kaolin.T4Full irrigation with 100% of AW + foliar application of 1.5 mM SiO_2_ NPs.T5Full irrigation with 100% of AW + foliar application of 3.0 mM SiO_2_ NPs.Group 2T6Irrigation with 80% of AW.T7Irrigation with 80% of AW + foliar application of 3% kaolin.T8Irrigation with 80% of AW + foliar application of 6% kaolin.T9Irrigation with 80% of AW + foliar application of 1.5 mM SiO_2_ NPs.T10Irrigation with 80% of AW + foliar application of 3.0 mM SiO_2_ NPs.Group 3T11Irrigation with 60% of AW.T12Irrigation with 60% of AW + foliar application of 3% kaolin.T13Irrigation with 60% of AW + foliar application of 6% kaolin.T14Irrigation with 60% of AW + foliar application of 1.5 mM SiO_2_ NPs.T15Irrigation with 60% of AW + foliar application of 3.0 mM SiO_2_ NPs.

### 3.3. Measurements

#### 3.3.1. Growth and Yield Traits

About 90 days following seeding, leaf and plant samples were taken to examine their growth and physiological and biochemical properties. In contrast, 2 m^2^ of maize was cut down when it reached maturity in order to analyze the yield and associated characteristics (plant height (cm), ear diameter (mm), ear length (cm), number of grains per ear^−1^, 100-grain weight (g), and grain yield (kg ha^−1^)).

#### 3.3.2. Photosynthetic Characteristics

A SPAD chlorophyll meter was used to measure the amount of chlorophyll SPAD in the maize leaves [32]. The net photosynthetic rate (PN), stomatal conductance (gs), intercellular CO_2_ concentration (Ci), and transpiration rate (E) were measured in the extended upper portion of the plant leaves in each treatment using the portable photosynthetic method.

#### 3.3.3. ROS Indicators

##### Lipid Peroxidation

Lipid peroxidation was employed to quantify the degree of lipid oxidation, and the techniques of Hernández and Almansa [33] were used to examine the malondialdehyde (MDA) level. Fresh maize plant leaves were centrifuged at 12,000× *g* shortly after being macerated in trichloroacetic acid (TCA). The supernatant was then submerged in water for 30 min before being subjected to thiobarbituric acid. Following cooling, the optical absorbance of the samples was assessed at 532 nm.

##### Hydrogen Peroxide

Leaf samples were extracted using 5% TCA and then centrifuged at 11,500× *g* for 15 min afterward. After treatment with 10 mM phosphate buffer (pH 7.0) and 1 MKI, the absorbance at 390 nm was determined [34].

##### Superoxide Anion

The superoxide anion (O_2_) concentration in the leaves was determined as per Jabs et al. [35] by removing them from a phosphate solution. First, a short incubation in hydroxylamine hydrochloride was applied to the extract. After 20 min of incubation, sulphanilamide and -naphthyl were added, and the optical density was then determined spectrophotometrically at 530 nm.

##### Hydroxyl Radical

The hydroxyl radical (OH) concentration was determined following the method of Halliwell et al. [36]. The last 1 mL of the reaction mixture included deoxyribose, 10^4^ mM EDTA, 20 mM KH_2_PO_4_ buffer (pH 7.4), 100 mM FeCl_3_, 1 mM H_2_O_2_, and 100 mM ascorbate. A measurement of the optical density at 532 nm was carried out after one hour of incubation at 37 °C.

#### 3.3.4. Total Proline and Phenol Content and Non-Antioxidant Enzymes

According to Bates et al. [37], ninhydrin acid, which is made from phosphoric acid and glacial acetic acid, was used to assess the amount of free proline in the leaf tissue. A sodium carbonate solution was used to determine the amount of free phenols in the leaf, and the Folin–Ciocalteu reagent was then measured at 765 nm [38]. Two grams of leaves were used to determine the ascorbic acid (AsA) content, which was then extracted using 5% (*w*/*v*) TCA and centrifuged at 15.600× *g* for five minutes. In a 1.0 mL reaction mixture of the supernatant, 10 mM DTT, 0.5% N-ethylmaleimide, and 10% TCA, the AsA level was checked immediately. Then, using a spectrophotometer and following the Jagota and Dani [39] procedure, the mixtures were incubated for 40 min at 40 °C, and the absorbance was measured at 532 nm.

The glutathione (GSH) content was determined by macerating fresh (100 mg) leaf tissue in a phosphate buffer (pH 8.0) and centrifuging it for 15 min at 3000× *g*. After a 10 min mixing period, 500 milliliters of supernatant were mixed with 5,5-dithiobis-2-nitrobenzoic acid, and the absorbance was measured at 412 nm [40].

#### 3.3.5. Determination of Antioxidant Enzymes 

Using H_2_O_2_ for consumption, the catalase activity was identified [41]. For three minutes, the H_2_O_2_ consumption was measured spectrophotometrically at 240 nm. The activity of superoxide dismutase (SOD) was measured using the technique described by Taniguchi et al. [42]. Nitro blue tetrazolium chloride (NBT) (2.25 mM), sodium carbonate (1.5 mM), methionine (200 mM), EDTA (3.0 mM), riboflavin (60 mM), and a phosphate buffer (100 mM) were all incorporated into 3 mL reaction mixtures (100 mM; pH 7.8). A spectrophotometer was used to measure the absorbance at 560 nm. Peroxidase (POX) was quantified according to the technique of Thomas et al. [43], using 0.2 mL of enzyme extract, 5.8 mL phosphate buffer (50 mM; pH 7.0), and 2 mL H_2_O_2_ (20 mM), and the absorbance was determined using a UV spectrophotometer (Model 6305, Jenway, cairo, Egypt) at 470 nm. A reduction in the absorbance at 265 nm was used as a spectrophotometric indicator of ascorbate peroxidase activity [44]. After oxidizing NADPH for one minute (extinction coefficient: 6.2 mM cm^−1^), the glutathione reductase activity was measured at 470 nm using a UV spectrophotometer (Model 6305, Jenway) [45].

#### 3.3.6. Determination of Mineral Contents in Grains

To analyze nitrogen (N), phosphorus (P), potassium (K), and silicon (Si) chemically, samples of the maize grains were crushed into a fine powder and dried for 48 h at 65 °C. Half a gram of dried grain samples was wet-ashed with a 3:1 solution of perchloric and sulfuric acids (H_2_SO_4_ + HClO_4_). The resulting acid-digested solution was then diluted with redistilled water to a final volume.

Following the instructions from Bremner [46], the amount of N in the acid digestion was determined using the Kjeldahl method. Next, the colorimetric method was used as in Page et al. [30] to determine the P content. Finally, the K content was determined photometrically using a flame photometer, according to Houba et al. [47]. Eraslan et al. [48] utilized blue silico molybdate to measure the Si content. The standard curve was created with silica salt. Combustion of the plant sample resulted in ash after three hours at 550 °C. Polycarbonate test tubes containing the ash were filled with 50 mL of 0.08 M H_2_SO_4_ and 2 mL of 40% hydrogen fluoride. An amount of 1.5 mL of this solution was mixed with 0.08 M H_2_SO_4_ and 20 g L^−1^ of ammonium molybdate to create the reagent combination; then, 1.5 mL of 0.2 M ascorbic acid was added to create the color. The absorbance was measured at 811 nm.

### 3.4. Statistical Analysis 

For the morphology portion of this research, a completely randomized design (CRD) was used with 15 treatments, 5 replications for morphology, and 3 repetitions for the biochemical analysis. SPSS (version 28.00; I.B.M. Corp, Armonk, NY, USA) was used for statistical analysis [49]. A two-way ANOVA used Fisher’s test with a 95% confidence level. The heat map displays the results of the Pearson correlation and discrimination analysis. Using the Origin Pro software, version 2018, a principal component analysis (PCA) was carried out. GraphPad Prism 8 was employed to create the charts.

## 4. Discussion

Water stress in arid and semi-arid climates can affect plant growth due to the limited supply of water to the roots, higher temperatures, and rapid transpiration rates [50]. Our findings showed that water stress reduced the growth and yield of maize. Furthermore, drought stress has been shown to reduce the growth of wheat plants [51]. Water stress may cause elongating cell deregulation due to the disruption in the water flow from the xylem to the elongating cells and decreases in growth-promoting hormones, mitosis during cell division, cell expansion, and cell elongation [52]. Water stress events occur around the world as a result of climate change [53]. As a result, new management will be required to address this issue using nanotechnology.

Reductions in morphological parameters and yield due to water stress have been reported for a variety of species, including wheat [21] and potato [54]. Reduced growth parameters may be due to increased MDA levels and the consequent shrinkage of cells, leaf growth reduction, decreased meristematic cell division, senescence acceleration, leaf drop, and leaf production blockage [22].

Furthermore, water stress can immediately impact the biochemical processes involved in photosynthesis and indirectly impact the entry of carbon dioxide into stomata, which close in response to drought. As a result, drought affects photosynthetic material transfer and photosynthesis is limited, reducing vegetative plant growth [55]. The positive effects of kaolin and SiO_2_ NPs have been reported in drought tolerance and the dry and fresh weights of different species such as maize. SiO_2_-NPs-induced growth stimulation has been linked to elevated antioxidative enzyme activities and photosynthesis under various environmental stress conditions [56]. These elements likely increase the production of assimilates and the activity of antioxidant enzymes such as SOD and CAT, reducing the negative effects of stress and promoting vegetative growth [57].

Due to the oxidative stress brought on by drought stress, the maize plant’s chlorophyll content, including its SPAD chlorophyll values and photosynthetic characteristics such as stomatal conductance, net photosynthetic rate, transpiration rate, and intercellular CO_2_ concentration, were decreased. This decrease might be the result of chlorophyll degradation and pigment photo-oxidation. Both sunflower and wheat plants have experienced similar outcomes [58,59]. In addition, drought stress causes plants to close their stomata to prevent water loss, reduce carbon transport [60], and decrease their Rubisco activity and ATP synthase [61].

The increase in photosynthetic pigments seen in maize plants treated with varying concentrations of kaolin and SiO_2_ NPs suggests that they improve the plants’ resistance to drought stress [62]. NPs, according to Bhattacharjee et al. [63], may cause chemical energy efficiency in photosynthetic systems. Furthermore, in Kataria et al. [64], the authors reported that nano-sized metal compounds bound to photosynthesis II (PSII) induced stable oxygen-evolving reactions, suggesting that light-driven electrons were transported from water molecules to quinone molecules. The author suggested that PSII conjugates might grow as photosensors and artificial photosynthetic devices. By accelerating the synthesis of CA and photosynthetic pigments, NPs accelerate the photosynthetic rate [65].

The cumulative impact of these altered processes may improve the capacity of the photosynthetic machinery in plants exposed to kaolin and SiO_2_ NPs during either non-drought stress or drought stress. These findings concur with those made previously by Behboudi et al. [66], who demonstrated that SiO_2_ NPs increased the Chl content of wheat leaves. The results show that MDA, H_2_O_2_, OH, and O_2_ levels were significantly greater in drought-stressed maize shoots than in unstressed plants. Through damaging DNA, proteins, and lipids, ROS lead to metabolic abnormalities and cell death. During drought stress, the MDA content significantly increased in maize plants [67] and sugar beet [68]. The increase in malondialdehyde levels may be explained by the fact that the plants’ enzyme activity is decreased during droughts [69]. The application of kaolin and SiO_2_ NPs to drought-stressed plants decreased MDA, H_2_O_2_, OH, and O_2_ levels compared to non-inoculated plants. By reducing MDA, H_2_O_2_, OH, and O_2_ levels, drought-induced oxidative stress and membrane damage in plants treated with kaolin and SiO_2_ NPs may be reduced compared to non-drought plants [54]. During drought stress, endogenous proline, phenol, AsA, and GSH were significantly increased when kaolin and SiO_2_ NPs were applied exogenously. Reactive oxygen species (ROS) have been shown to form uncontrollably at several locations in plants that have been exposed to environmental impacts, abiotic stress (drought, salt, and heavy metals), etc. [70]. Increased levels of ROS impede enzyme activity and cause oxidative damage to lipids, proline, phenol, AsA, and GSH [71]. Therefore, organelles have developed antioxidant defense mechanisms to save plant cells from oxidative harm by scavenging ROS [72]. 

Our results indicate that osmolytes accumulate more under drought conditions. During drought, heavy metal cells adjust their osmotic balance and suffer a decline in cell injury because of proline accumulation [73]. As a result of a reduction in water availability and photosynthesis, dry matter production decreases. By scavenging ROS and adjusting the pH of cells, proline, as a source of carbon and nitrogen, effectively protects structural molecules against denaturation due to drought stress [22]. A previous study indicated an enhanced accumulation of osmolytes, such as proline, in plants exposed to drought stress, such as trifoliate orange rootstock [74]. As a result of adding Si, particularly SiO2 NPs, more osmolytes were accumulated. Plants utilize proline as an osmolyte and nitrogen source to protect proteins from denaturation, and Si enhances nitrogen uptake and facilitates the production of soluble N complexes as proline [75].

In this experiment, drought-stressed maize plants had higher phenolic contents. Another study on grapevine leaves and roots exposed to drought [76] showed the same result. Certain phenolic compounds are increased in plant species exposed to water deficits, and the ultimate change is particularly dependent on the dominant phenol type. Experimental results supported these findings, and these differences in consequences can be explained by the types of abiotic stress (heavy metals, salinity, and drought) and biotic stress, their duration and intensity, the types of plant parts that were evaluated, or the stage of development of the plants [77]. A significant increase in phenolic content and antioxidant activity was observed after applying kaolin and Si NPs. The increase in non-enzymatic antioxidant substances, such as the phenolic compounds associated with Si implication, in plant metabolism is related to the Si-induced enhancement of abiotic stress (heavy metals, harmful microorganisms, and excessive fertilization) tolerance in plants [78]. Notably, phenolic compounds contribute to antioxidant activity, and there appears to be a remarkable correlation between antioxidant activity and phenolic content [79].

Moreover, treatment with SiO_2_ NPs enhanced endogenous GSH and AsA levels in strawberries under drought stress [22]. This may have been a preventative strategy given that a larger amount of GSH has been demonstrated to lessen oxidative damage efficiently and to squelch free radicals [54]. Moreover, GSH is essential for the ASC-GSH cycle [80], which may aid in regenerating the water-soluble antioxidant AsA and increase its levels in response to drought stress.

To allow the maize plant to survive drought stress, treatment with kaolin and SiO_2_ NPs may activate and control the activities of several antioxidant enzymes, such as CAT, POX, APX, GR, and SOD. Due to the disruption in cell homeostasis brought on by environmental stress such as drought, excess ROS were created in plants. When the amount of ROS produced surpasses the capacity of the cell’s immune system, oxidative stress occurs, which leads to reduced enzyme activity, nucleic acid oxidation, protein oxidation, the activation of the major apoptotic pathway, MDA, and cell death [81]. Antioxidant enzymes may thereby prevent injury by removing excess ROS produced during drought stress. Several studies have noted that increasing the expression of different antioxidant enzymes may improve stress resistance. As the first and most efficient line of defense against ROS in these areas, SOD is a crucial antioxidant enzyme that safeguards against abiotic and biotic stress in chloroplasts, mitochondria, peroxisomes, endoplasmic reticulum, cell walls, apoplast, and plasma membranes [82]. In response to drought stress, SOD activity has increased in chickpeas. In addition, SOD is a crucial antioxidant enzyme that scavenges O_2_, OH, and H_2_O_2_ in the Haber–Weiss process [83]. 

By increasing the activities of the APX, CAT, GR, SOD, and POX enzymes, the maize plant has thus been protected against free-radical-induced membrane malfunction. Due to improvements in lignin and other antioxidant chemicals that reduce formation, the significantly higher POX activity in maize plants treated with kaolin and SiO_2_ NPs may be associated with increased sensitivity to drought stress [66]. Ghasemi et al. [84] found that POX activity in maize was increased under drought stress. Low H_2_O_2_ levels may be related to an increase in CAT activity in maize leaves grown under drought stress [85]. Moreover, H_2_O_2_, a powerful and harmful oxidizing agent, was converted into H_2_O and O_2_ by CAT and APX [86]. Zahedi et al. [22] discovered that treatment with kaolin and SiO_2_ NPs induced substantial variations in the activity of APX in maize plants under drought stress. After the exogenous administration of SiO_2_ NPs, GR activity increased; as a result, it may be engaged in recycling and raising endogenous GSH [87]. 

The exogenous foliar application of kaolin and SiO_2_ NPs also improved GR activity in maize plant shoots. This suggests that GSH can maintain the structure of biological macromolecules and defend the sulfhydryl (-SH) groups of enzymes and structural proteins from oxidation [88]. Moreover, the elevated Si concentration enhanced the AsA-GSH cycle, elevating GR and APX activities while increasing the amount of AsA. ROS were eliminated due to these enzymes’ combined effects [89]. 

An increase in GSH production and/or degradation has been linked to higher GSH contents in plants that can withstand heat and drought [90]. In addition, the exogenous 3 mM SiO_2_ NPs were more effective than 6% kaolin in promoting the activities of CAT, GR, SOD, POX, and APX, suggesting that exogenous SiO_2_ NPs stimulate the AsA–GSH cycle more effectively [82,91]. Drought stress greatly lowered the P, N, and K contents in maize grains compared to plants without drought stress. Conversely, the N, P, and K contents in the grains of drought-stressed maize plants were dramatically increased via the exogenous application of kaolin and SiO_2_ NPs. 

Kim et al. [92] revealed that nutrient absorption decreased during drought stress due to a restriction in hydraulic conductivity, a reduction in root length, root branching, and an increase in root thickness. Due to the limited water supply, there was a significant reduction in N, P, and K concentrations in maize grains. A result of this was limited nutrient uptake because transpiration was reduced, ions were immobilized, membrane permeability was reduced, and the roots were not able to absorb nutrients [93]. It has been reported that N, P, and K uptake decreases with an increased duration of water deficit [94]. Insufficient soil moisture and osmotic stress are two factors that contribute to decreased nutrient uptake. This is due to the reduced solubility of nutrients around root hairs [95]. Under drought stress, the foliar spraying of kaolin and SiO_2_ NPs increased NPK and Si concentrations, which increased nitrification and water retention in the rhizosphere. A water deficit decreases P activity and fixation in alkaline soils. Although kaolin and SiO_2_ NPs increased root function under water stress and alleviated osmotic pressure, foliar application of nano-silica augmented P and K uptake. Additionally, kaolin and SiO_2_ NPs could prevent leaf water depletion and increase K absorption in the leaves. Under water deficit conditions, Amer and El-Emary [96] reported that maize’s response to Si increased K, P, and N concentrations. Using a Si application in stressed plants, Zahedi et al. [22] found that Si promoted P uptake, while Ali et al. [97] found that Si application mediated K accumulation in stressed plants. By enhancing osmoregulatory substances and antioxidant activity in plant tissues, Liu et al. [98] demonstrated that Si application increases the concentration of different mineral elements, including K.

Wheat harvest index and nitrogen use efficiency were observed to be increased by Si availability in another study. Ocvirk et al. [99] reported that sunflower seed priming provides energy and nutrients to induce drought tolerance. Applying exogenous Si enhances NPK concentrations in maize seedlings experiencing water stress [22].

## 5. Conclusions

The detrimental effects of drought on growth, biochemical features, and yield attributes were considerably decreased when kaolin and SiO_2_ NPs were applied to maize plants. The alleviation of drought stress brought on by the kaolin and SiO_2_ NPs may be attributable to the suppression or minimization of drought accumulation in maize plants, which lowers the degree of ROS-induced damage to the membrane system. Moreover, the absorption of vital nutrients, including nitrogen, phosphorous, and potassium, which are necessary for plant development, was boosted by the kaolin and SiO_2_ NPs. As a result, the activity associated with photosynthesis was improved by the kaolin and SiO_2_ NPs. Additionally, to provide maize plants with the best defense against drought stress, kaolin and SiO_2_ NPs controlled the activity of stress enzymes. This research may also highlight the potential processes behind the resistance to drought toxicity generated in maize by kaolin and SiO_2_ NPs. Our findings demonstrated that SiO_2_ NPs at 3 mM were the most effective treatment for decreasing the detrimental effects of drought and enhancing growth, yield characteristics, biochemical and physiological characteristics, and the concentrations of N, P, K, and Si, followed by kaolin at 6%.

## Figures and Tables

**Table 1:** Physical and chemical properties of soil at the experimental site before planting (average of the two seasons).

Soil Property	Soil Layer (cm)			
	0–15	15–30	30–45	45–60
Particle size distribution:				
Coarse sand (%)	4.78	4.81	5.11	5.39
Fine sand (%)	74.35	75.14	75.58	75.99
Silt (%)	6.55	6.52	6.44	5.98
Clay (%)	14.32	13.53	12.87	12.64
Texture class	Sandy loam	Sandy loam	Sandy loam	Sandy loam
Field capacity $(\theta_{\mathrm{FC}}$, %)	14.45	14.14	13.96	13.84
Permanent wilting point ($\theta_{\mathrm{PWP}}$, %)	6.11	5.99	5.84	5.71
Available water (AW, %)	8.34	8.15	8.12	8.13
Bulk density (Mg m^−3^)	1.46	1.65	1.71	1.74
Total porosity (%)	44.91	37.74	35.47	34.34
pH (1:2.5 soil/water suspension)	7.88	7.87	7.91	7.94
EC_e_ (soil paste extract, dSm^−1^)	1.65	1.63	1.50	1.35
Organic carbon (g kg^−1^)	2.69	2.66	2.54	2.44
Organic matter (g kg^−1^)	4.63	4.58	4.37	4.20
CaCO_3_ content (g kg^−1^)	21.10	22.35	23.15	23.87
Soluble cations (mmolc L^−1^):				
Ca^2+^	2.15	2.09	1.97	1.85
Mg^2+^	2.77	2.69	2.59	2.37
Na^+^	10.91	10.86	9.85	8.71
K^+^	0.67	0.65	0.63	0.59
Soluble anions (mmolc L^−1^):				
CO_3_^2−^	0.00	0.00	0.00	0.00
HCO_3_^−^	2.97	2.91	2.87	2.75
Cl^−^	8.98	8.85	7.79	6.82
SO_4_^2−^	4.55	4.53	4.38	3.95

**Table 2:** Temperature (°C), wind speed (m s^−1^), relative humidity (%), and surface pressure (kPa) of the experimental site during the summer seasons of 2021 and 2022.

Month	Temperature (°C)		Wind Speed (m s^−1^)		Relative Humidity (%)	Surface Pressure (kPa)
	Max	Min	Max	Min		
2021						
May	42.01	11.83	10.33	0.40	50.69	99.77
June	42.12	15.25	10.20	0.38	41.50	99.48
July	41.68	18.37	8.44	0.44	42.88	99.12
August	42.68	20.30	8.48	0.80	45.38	99.15
2022						
May	41.67	14.90	9.29	0.32	36.69	99.64
June	41.45	15.86	8.85	1.73	41.38	99.62
July	43.00	20.12	9.15	1.01	41.12	99.16
August	44.01	20.37	8.41	0.49	42.88	99.26

## Data Availability

Not applicable.
